# Evaluation of a Chaotrope and Kosmotrope in the Multivariate Optimization of PHW-ATPE of Solasodine from Leaves of *Solanum mauritianum*

**DOI:** 10.3390/molecules27175547

**Published:** 2022-08-29

**Authors:** Tebogo Mphatlalala Mokgehle, Ntakadzeni Edwin Madala, Nikita Tawanda Tavengwa

**Affiliations:** 1 Department of Chemistry, Faculty of Science, Engineering and Agriculture, University of Venda, Private Bag X5050, Thohoyandou 0950, South Africa; 2 Department of Biochemistry, Faculty of Science, Engineering and Agriculture, University of Venda, Private Bag X5050, Thohoyandou 0950, South Africa

**Keywords:** solasodine, *S. mauritianum*, response surface methodology, PHW-ATPE

## Abstract

A hyphenated pressurized hot water—aqueous two-phase extraction (PHW-ATPE) method was applied to extract solasodine from *Solanum mauritianum* (*S. mauritianum*). A central composite design (CCD) was applied to determine the optimal conditions for the extraction of solasodine. The parameters evaluated included the percentage concentration of salt (NaCl or Na_2_CO_3_) and temperature. The fit of the central composite design response surface model for PHW-ATPE to the data generated a model with a good quadratic fit (R^2^ = 0.901). The statistically significant (*p* < 0.05) parameters, such as the linear and quadratic effects of the concentration of salt (%) powder, had a significant impact on the extraction of solasodine. The application of multiply charged salts such as Na_2_CO_3_ (kosmotrope) was shown to be a comparably better extractant of solasodine than NaCl (chaotrope) due to the salting-out effect. The optimized conditions for extraction of solasodine with NaCl or Na_2_CO_3_ were a temperature of 80 °C at a salt concentration of 20%. The maximum extraction of solasodine was 300.79 mg kg^−1^ and 162.34 mg kg^−1^ for Na_2_CO_3_ and NaCl, respectively.

## 1. Introduction

Over the past several decades, the application of chemistry in the industry has been directed at the use of environmentally friendly approaches [1,2,3,4]. The current adoption of environmentally friendly methods has been inspired by the 12 Principles of Green Chemistry as initiated by Anastas and Warner (1998) [5]. This concept aimed to revolutionize chemistry by employing innovative scientific solutions to solve environmental dilemmas. Two of the principles dwelt on using safer solvents that are degradable. This is fundamental, as the extent of environmental impact is affected by the type of solvent used. Furthermore, the application of green solvents affects the way natural resources are harvested, energy usage, and emissions to air and water from the production and use of solvents, transportation, and disposal or recycling [6,7,8]. Hence, water as a potentially green extraction solvent fits this category well, as it is nontoxic to health and the environment and is the safest, abundant, and least expensive solvent.

PHWE (also called subcritical water extraction or superheated water extraction) is based on the use of water subjected to high enough temperatures (usually above its boiling point) and pressures to keep the water in its liquid state [9]. Therefore, water that remains a liquid at temperatures above its boiling point (100 °C, 0.1 MPa) and below its critical point (374 °C, 22.1 MPa) is utilized as a solvent in PHWE [9,10,11]. The principle of PHWE is guided by the physiochemical properties of water. Water is highly polar with a high dielectric constant (ε) of 80 at room temperature and atmospheric pressure because of its extensive hydrogen-bonded structure [12,13]. Traditionally, water is not known to dissolve non-polar compounds at room temperature. However, as the temperature of the water is increased, there is a resultant decrease in its permittivity, viscosity, and surface tension but an increase in its diffusivity characteristics. Similarly, at elevated temperatures, the dielectric constant of water decreases from ε = 80 at 25 °C to ε = 27 at 250 °C and 50 bar [9,10]. Under these conditions, water has a dielectric constant compared to other organic solvents, such as methanol (ε = 33) and ethanol (ε = 24) at 25 °C. Additionally, water can dissolve a wide range of medium and low polarity analytes [12,13].

Lately, miniaturization for separation processes has become a crucial technique in various disciplines. These include biological engineering, pharmacy, environmental detection, and laboratory analysis [14,15]. For instance, Rodrigues et al. [14] applied a miniaturization technique in the form of micro-QuEChERS for the quantification of psychotropic drugs in postmortem blood samples. In another study, Lendor et al. [15] investigated solid phase microextraction (SPME), which was applied for the extraction of neurotransmitters from brains [15]. Miniaturization has also been applied in the detection of pollutants in the environment, for example, Abaroa-Pérez et al. [16] applied solid-liquid-liquid microextraction (μSLLE) for the enrichment of polyaromatic hydrocarbons (PAHs) and polychlorinated biphenyls (PCBs) in microplastics located in coastal areas. Some of the many advantages miniaturized extraction offers are improved heat and mass transfer, which has been reported to result in enhanced separation efficiencies [17,18,19].

Given the potential water has as an extraction solvent, this study is a follow-up to the work conducted by Mokgehle et al. [20] where ATPE was only used for the extraction of solasodine from *Solanum mauritianum* (*S. mauritianum*), which is an invasive species [20]. Furthermore, PHW-ATPE could be a gateway for time-efficient, robust, and energy-efficient extraction techniques that could enrich bioactive metabolites from a variety of organic waste materials such as weeds, eggshells, apple cores, and coffee grounds. For the first time, this work applied a hyphenated method involving PHW-ATPE in an attempt to enhance the extraction of solasodine from *S. mauritianum*. Solasodine has attracted attention due to its impressive anticancer activity and insecticide properties, hence it is a sought-after metabolite [21,22,23,24,25]. Firstly, in this work, two salts were investigated to aid the PHW-ATPE process; the doubly charged (Na_2_CO_3_, which is the kosmotrope) and the singly charged (NaCl, which is the chaotrope) to determine which of the two would better extract solasodine. Thereafter, the extracts were analyzed on the UHPLC-QTOF-MS and modeled using computational methods involving central composite design (CCD) and response surface methodology (RSM). This statistical approach is beneficial as it minimizes the number of experiments to be performed, reducing the workload and the time taken to conduct a set of experiments [26,27]. The possible synergistic effect of salting-out with water pressure and temperature was also investigated in this work. This hyphenated environmentally friendly extraction technique, which only uses water as an extraction solvent, could potentially be utilized on a commercial scale.

## 2. Materials and Methods

### 2.1. Chemicals and Reagents

**Figure 1:**
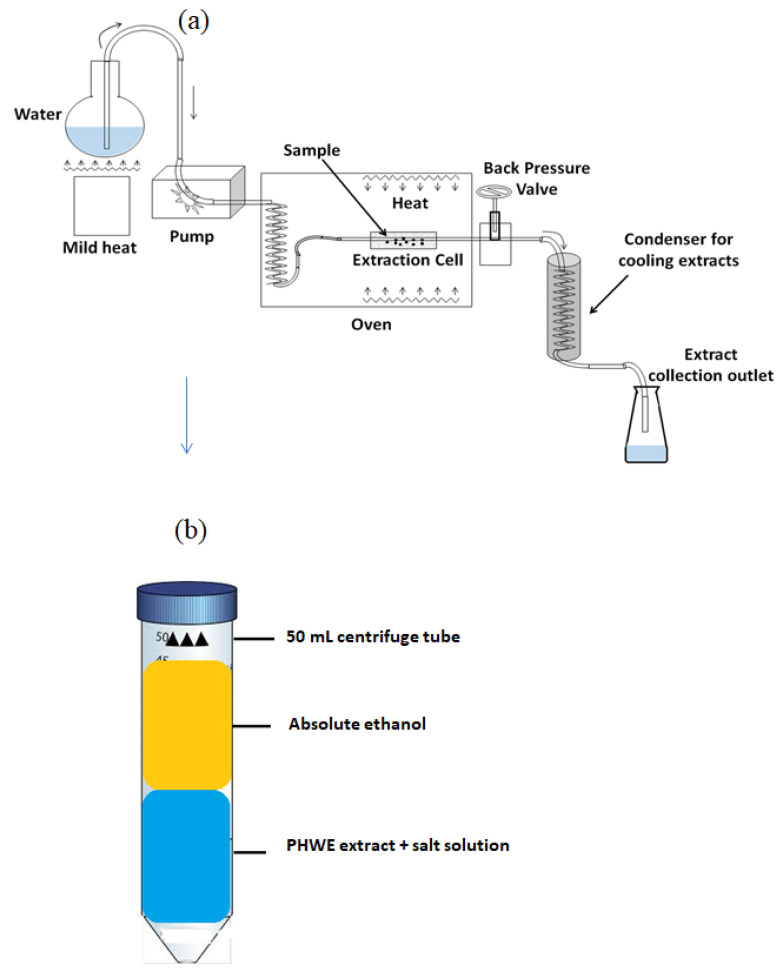
(**a**) A PHWE extraction system was consisting of the inflowing water propelled by a pump into the extraction cell. The metabolites contained in water were then cooled within the condenser before being collected in the Erlenmeyer flask. (**b**) A PHW-ATPE set-up.

The salts NaCl (anhydrous > 99% purity), Na_2_CO_3_ (anhydrous > 99% purity), and ethanol (99% CP) were purchased from Associated Chemical Enterprises (Johannesburg, South Africa) and Sigma-Aldrich (Johannesburg, South Africa). Ultra-pure water (0.005 µS, 18 mΩ) using a Direct-Q 5UV distiller (Massachusetts, United States of America) was applied to prepare the salt solutions. The extraction was performed on a DIAB MX-RL-Pro dragon shaker. A makeshift laboratory-scale PHWE unit was used for the extraction of phytochemicals (Figure 1a,b). The system consisted of an HPLC pump (Waters 6000 fluid controller, Waters Corporation, Manchester, UK), stainless steel extraction cell (70 × 30 mm, approximately 20 mL) fitted with a metal frit, i.e., filter (3/8 in. diameter, 1/32 in. thickness and 2.0 μm pore size), refurbished GC 600 Vega Series 2 oven (Carlo Erba Instruments, Milan, Italy) with an automatic temperature control unit, stainless steel tubing (1.58 mm in outer dimension (OD) and 0.18 mm inner dimension (ID), back-pressure valve (Swagelok, Johannesburg, South Africa), and a collection flask. Chromatographic separation of the metabolites in the extracts was performed using a reverse-phase Shim-pack Velox C18, 2.1 × 100 mm, 2.7 µm with a serial number of 227-32009-03 (COU, Columbia, NY, USA). The UPLC was connected to a Shimadzu 9030 LC, qTOF-MS detector (Shimadzu, Kyoto, Japan). The solvents used for the chromatographic runs were methanol and formic acid, which were purchased from Romil Pure Chemistry (Cambridge, UK).

### 2.2. Sample Collection, Preparation, and ATPE

The leaves of *S. mauritianum* were collected from a farming district in Phiphidi, on the outskirts of Thohoyandou, South Africa. The plants were air-dried until a constant weight was obtained, and the leaves were ground into a fine powder with a blender at 2000 rpm and stored in glass containers. The containers were covered in paper bags to prevent light penetration. For the extraction, 3 g of ground leaf powder was mixed with 1.5 g of diatomaceous earth (Sigma, Munich, Germany), a dispersing agent, and placed inside the extraction cell maintained at different oven temperatures of 80, 120, and 200 ± 1 °C. The solvent was delivered at a constant flow rate of 10 mL min^−1^, and a pressure of 2500 ± 300 pa was maintained using the back-pressure valve. Extracts were collected in a falcon tube up to the 150 mL mark through an outlet coil immersed in a cooling water bath. The PHWE extracts (10 mL) were added to salt solutions containing 20, 35, and 50% (*w*/*v*) of NaCl (kosmotrope) or Na_2_CO_3_ (chaotrope). This solution was placed on the dragon shaker for 24 h, rotating at 70 revolutions per minute (rpm). After that, absolute ethanol (10 mL) was added, resulting in a PHW-ATPE system (Figure 1b). The extracts were filtered using a 0.22 μm nylon syringe filter into a 2 mL HPLC capped vial and preserved at −20 °C before analysis on the UPLC-QTOF-MS for detection of solasodine.

### 2.3. Chromatographic and Mass Spectrometry Conditions

Solasodine was separated using a Shimpack C18, 2.1 × 100 mm, 2.7 µm column from Shimadzu (Honeydew, South Africa). The column was maintained at 40 °C at a flow rate of 0.4 mL min^−1^ and the injection volume was 5 µL. Mobile phase A was 0.1% formic acid in ultrahigh purity water (*v*/*v*) and mobile phase B was 0.1% (*v*/*v*) formic acid in methanol. For enhancement of peak capacity and resolution, the elution gradient was 10% B for 2 min, ramped to 90% in 1 min and kept at 90% B for 1 min, and returned to 10% B for 1 min. The column was allowed to re-equilibrate for 1 min at 10% B.

An UPLC-QTOF-MS 9030 mass spectrometer (Shimadzu, Japan) was used for all mass spectral measurements. The mass spectrometer was equipped with an electrospray interface (ESI) operating in positive mode. ESI parameters were optimized for solasodine by direct infusion of standard solutions into the mass spectrometer. The mass spectrometer was operated in the multi-reaction monitoring (MRM) mode to confirm the identity of solasodine. This was achieved by selecting specific precursor to product ion transitions for each solasodine based on MRM transitions. The optimum conditions were as follows: high-purity nitrogen (N_2_) at a flow rate of 3 L min^−1^ was used as the nebulizing gas. High-purity nitrogen was also used as a heat gas at a flow rate of 10 mL min^−1^. The interface and desolvation temperatures were 300 and 526 °C, respectively. The collision energy was 30–60 eV. The interface voltage (at negative ionization) was 4.5 KV. The desolvation line (DL) temperature was 250 °C. The DL (bias) voltage of 2.5 V was relative to the tuning file. The heat block temperature was 400 °C at a detector voltage of 1.8 KV. High-purity nitrogen (N_2_) was used as the nebulizing and drying gas. The optimum parameters were as follows: drying gas temperature, 250 °C and the drying gas flow was 10 L min^−1^. For chromatographic identification, a Shimadzu 9030 LC instrument (Shimadzu, Japan) was used. The instrument consisted of an autosampler, a column thermostated at 50 °C, and a binary pump. Lab solutions software was used to control the LC-MS/MS instrument and for data acquisition, and the mass range was *m*/*z* 100–1000.

### 2.4. Statistical Analysis

The central composite design response surface model (CCD RSM) was fitted to experimental data to obtain the relationship between factors and optimize the response of Z (solasodine yield) concerning A (time), B (mass of plant powder) using Minitab 17 (UK). A two-level full factorial CCD was designed, a total of 36 experimental runs (including two repetitions) were designed, 3 numerical factor levels for the concentration of salt (20, 35, and 50%), temperature (80, 140, and 200 °C), and two categorical factor levels for salts, which included the chaotrope (NaCl) and kosmotrope (Na_2_CO_3_).

The model parameters and model significance were determined at *p* < 0.05. The model’s fitness was determined by evaluating the coefficient of regression (R^2^) obtained from the analysis of variance (ANOVA). The model fit generated the response surface that defined the behavior of the response variable. By utilizing these plots, the optimized ranges for each factor that led to the highest response (i.e., concentration of solasodine) can be extracted.

The interaction between the various parameters studied and its resultant effect on the extraction of solasodine (mg kg^−1^) was fitted to experimental data by using a statistical multiple regression approach method of least square (MLS), which resulted in the lowest possible residual 26. The model parameters and model significance were determined at *p* < 0.05. The model’s fitness was determined by evaluating the coefficient of regression (R^2^) obtained from the analysis of variance (ANOVA). The model fit generated the response surface that defined the behavior of the response variable. Through these plots, the optimized ranges for each factor that led to the highest response (i.e., concentration of solasodine) can be extracted [28,29].

## 3. Results and Discussion

### 3.1. MRM Quantification of Solasodine Based on the 414 → 396 Transition

**Figure 2:**
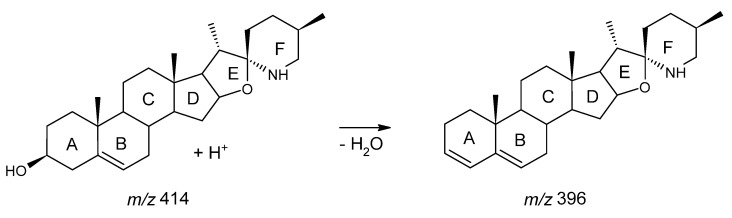
The fragmentation of solasodine (*m*/*z* = 414) to [solasodine—H_2_O] (*m*/*z* = 396) after the loss of water.

This plant is also a rich source of anticancer and antifungal metabolites such as solasodine and solasodine glycosides [30,31]. The ATPE technique was performed by assessing the various factors shown in Table 1. The presence of solasodine has been reported in *S. mauritianum* and other species within the *Solanum* genus [30,31,32,33]. Using a sensitive and robust tandem mass spectroscopy approach (UHPLC-qTOF-MS) with settings presented in the work performed by Senizza et al. [33], it was possible to successfully fingerprint solasodine fragmentation based on the *m*/*z* 396 product ion (Figure 2). Thereafter, based on the 414 → 396 transition within the MRM method, solasodine was quantified, as shown in Table 1.

### 3.2. Fit Statistics and Pareto Chart of Parameter Main Effects and their Interactions Produced from ANOVA and Resultant Box Plots

**Figure 3:**
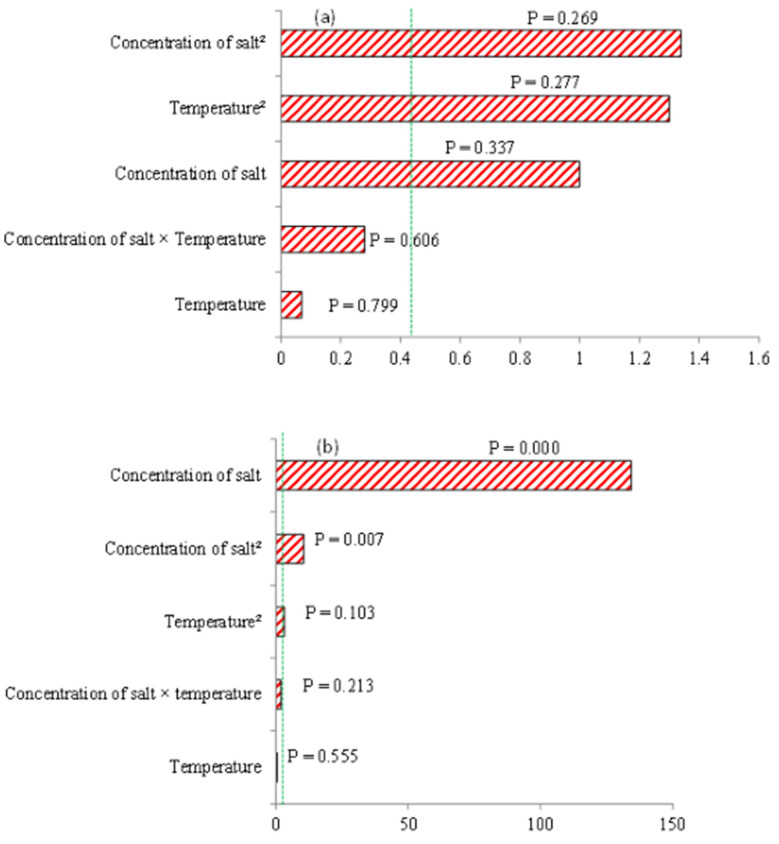
Pareto chart of various solasodine extraction conditions at *m*/*z* 414 → 396 in the presence of (**a**) NaCl and (**b**) Na_2_CO_3_.

The model fitted had a quadratic fit with *p*-values less than 0.0001, indicating that the model terms are significant. The probabilities for the concentration of salt and temperature for NaCl and Na_2_CO_3_ were *p* = 0.269 and *p* = 0.799 and *p* = 0.000 and *p* = 0.55, respectively, as shown in the Pareto charts in Figure 3a,b). This indicated that the linear effect of the concentration of Na_2_CO_3_ was a significant (*p* < 0.05) model term and was an adequate predictor of the experimental values obtained (Figure 3b). Similarly, the quadratic effect of the concentration of Na_2_CO_3_ was significant (*p* = 0.005) (Figure 3b). The rest of the model terms for Na_2_CO_3_ and NaCl were insignificant (*p* > 0.05), which also included the linear and quadratic effects of temperature (Figure 3a,b). Similar observations were reported by Gbashi et al. [34] on the insignificance of temperature (*p* > 0.05) during PHWE of dicaffeoyl quinic acids from *Bidens Pilosa*. The lack of fit of the F-value was observed to be 1.71, which indicated that the lack of fit was not significant relative to the pure error. The non-significant lack of fit was desirable. The goodness of fit between the experimental and predicted values was R^2^ = 0.901.

**Figure 4:**
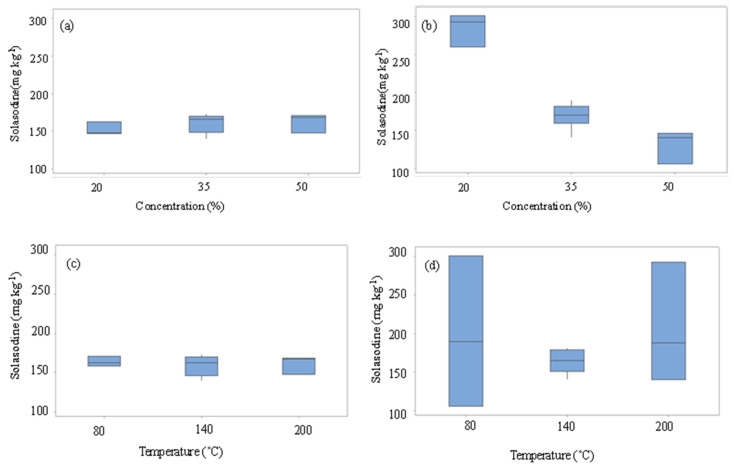
Box-and-whiskers plots evaluating the effect of ‘Concentration of salt on (**a**) NaCl, (**b**) Na_2_CO_3_ and the effect of ‘Temperature’ on (**c**) NaCl, and (**d**) Na_2_CO_3_ on the extraction of solasodine from leaves of *S. mauritianum*.

**Figure 5:**
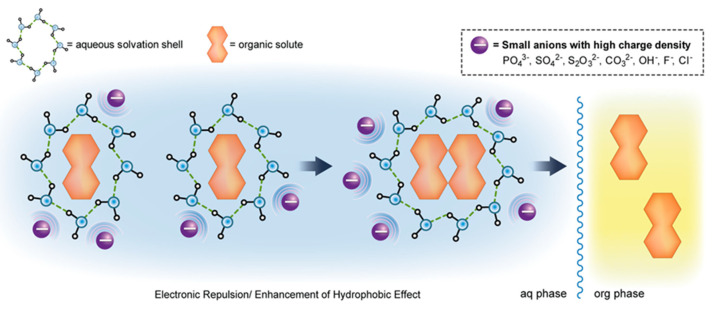
Molecular forces that dictate the aqueous solubility of organic solutes [33].

Similarly, in Figure 4a–d, the box-and-whisker plots of the effect of concentration and temperature on the PHW-ATPE extraction of solasodine from leaves of *S. mauritianum*. From Figure 4a–d as the % concentration of salt was increased for Na_2_CO_3_, a decrease in solasodine extracted was observed. At the same time, there were no notable changes in solasodine (≈160 mg kg^−1^) concentration, as salt concentration was varied for NaCl (Figure 4a,b). The highest extraction of solasodine when the variation of salt concentration was evaluated was approximately 300 mg kg^−1^. This indicated that a doubly charged anion, CO_3_^2−^, was more efficient than a singly charged ion, Cl^−^, during the salting-out of solasodine at low % salt concentrations (Figure 4b), as most of the salt was dissolved into the solution. However, higher concentrations of salt led to a super-saturated solution, which led to its precipitation from the solution, reducing the salting-out efficiency at 50% salt concentration for Na_2_CO_3_ in particular. The relatively higher solasodine extraction capability of Na_2_CO_3_ in comparison to NaCl can be explained based on the Hoffmeister series where CO_3_^2−^ has a better solute (solasodine) precipitation ability than Cl^−^. This trend is due to the divalent carbonate ion having a higher charge density than the monovalent chloride ion (Figure 5) [33,35,36,37,38]. The disruption of the solvation shell by the anions is followed by the salting-out of organic solutes (solasodine) from the aqueous phase to the organic phase [33]. The divalent property of the carbonate ion resulted in the salting-out of solasodine occurring to a greater extent compared to the monovalent chloride ion. The more enhanced the salting-out process is, the greater the extraction of the solute (solasodine) from the aqueous phase into the ethanol (extractant phase). Similarly, Bulgariu et al. [39], Neves et al. [40], and Li et al. [41] reported on the better salting-out capacity of SO_4_^2−^ compared to Cl^−^.

Generally, temperature variation did not have a significant effect (*p* < 0.05) on solasodine extracted when NaCl was used as an extraction agent. The kosmotrope Na_2_CO_3_ was more responsive to temperature changes, with the highest extraction achieved at 80 °C. This probably implies that Na_2_CO_3_ does not require higher temperatures for efficient extraction of solasodine but can be performed at lower temperatures, which is recommended in green chemistry. Furthermore, the application of Na_2_CO_3_ in PHW-ATPE demonstrated that extraction of solasodine was mainly driven by the salting-out process rather than the temperature (Figure 3b). Furthermore, both salting-out and temperature seemed to be insignificant in the presence of the NaCl extraction agent.

### 3.3. Chromatographic Profile of MRM-Based Quantification of Solasodine

**Figure 6:**
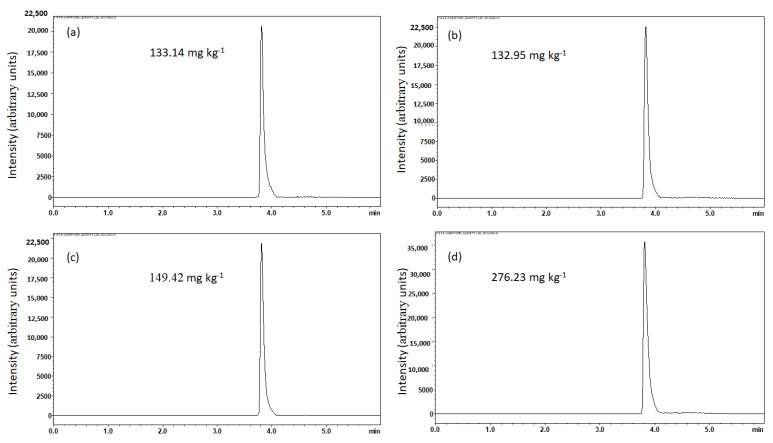

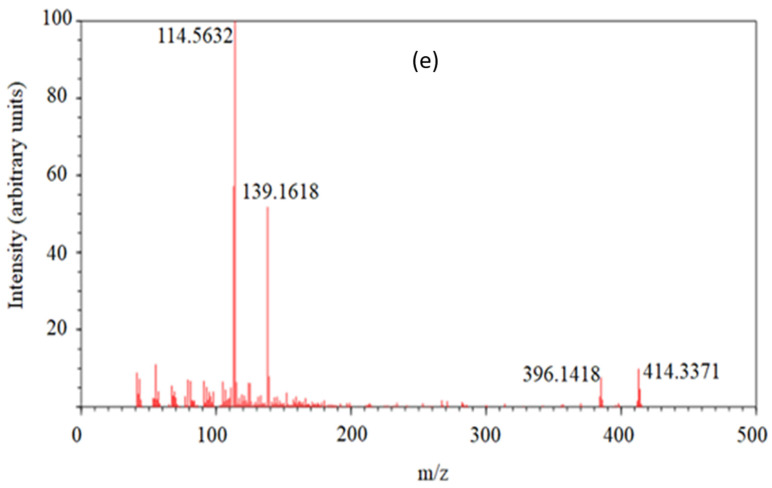
Chromatogram of the lowest extracted concentration of solasodine in (**a**) NaCl and (**b**) Na_2_CO_3_, and highest extracted concentration of solasodine (**c**) NaCl and (**d**) Na_2_CO_3_ for running 1. (**e**) mass spec of solasodine.

Chromatograms showing the lowest and highest concentrations of solasodine (mg kg^−1^) obtained in the presence of NaCl and Na_2_CO_3_ were applied as shown in (Figure 6a–d). From these chromatograms, an MRM transition of solasodine at *m*/*z* 414 → 396, following the loss of water (Figure 2), is observed at a retention time of 3.825 min (Figure 6a–d). The fragmentation profiles depicting the daughter ions of solasodine are contained in Figure 6e. The other daughter ions at *m*/*z* 139 and *m*/*z* 114 indicated the presence of unmodified F-rings (Figure 2) [20]. As stated before in Figure 2, solasodine was dehydrated from the precursor ion at *m*/*z* 414 to the product ion at *m*/*z* 396 (Figure 2). The experimental results indicate that dehydration of solasodine generally seems to be favored at extraction parameters involving lower percentage concentrations of salts and lower extraction temperatures (Figure 4b). Higher temperatures seem to have led to solasodine degradation. These conditions resulted in the extracted solasodine concentrations of 149.42 mg kg^−1^ and 276.23 mg kg^−1^ when both NaCl and Na_2_CO_3_ were applied to aid extraction, as shown in Figure 6c,d, respectively. This also concurs with the significant effect of the concentration of salt, particularly Na_2_CO_3_, as seen in the Pareto chart in Figure 3b, and the box plots in Figure 4.

### 3.4. Optima Obtained from Response Surface Equations with the Aid of NaCl or Na_2_CO_3_

**Figure 7:**
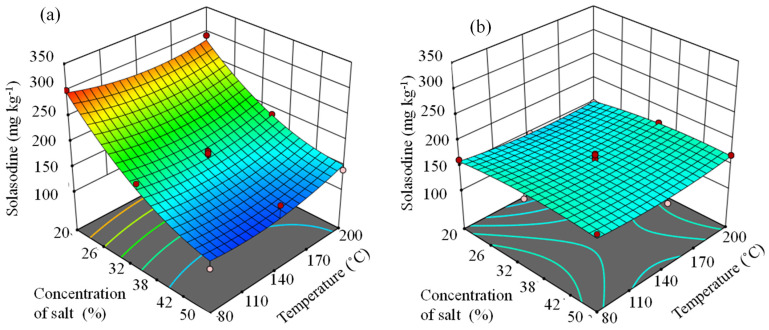
Response surfaces evaluating the effect of time and mass of plant powder in the presence of (**a**) Na_2_CO_3_ and (**b**) NaCl on the extraction of solasodine.

The response equations corresponding to NaCl and Na_2_CO_3_ are depicted as Equations (1) and (2), respectively. The responses to the bivariate interaction between time and mass on solasodine extraction are illustrated in Figure 7. In this case, Z, the concentration of solasodine was the dependent variable (solasodine concentration) and x (concentration of salt (%)) and y (temperature (°C)) was the independent variables. From the quadratic fit of r^2^ = 0.901 as reported in Section 3.2, the following Equations (1) and (2) were obtained:

z (x,y) = 167.79 + 2.35 × −0.76 y − 0.036 × 2 + 0.0021 y2 + 0.0035 xy (1)

z (x,y) = 649.18 − 16.205 × −1.63 y + 0.1346 × 2 + 0.0046 y2 + 0.0118 xy(2)

**Figure 8:**
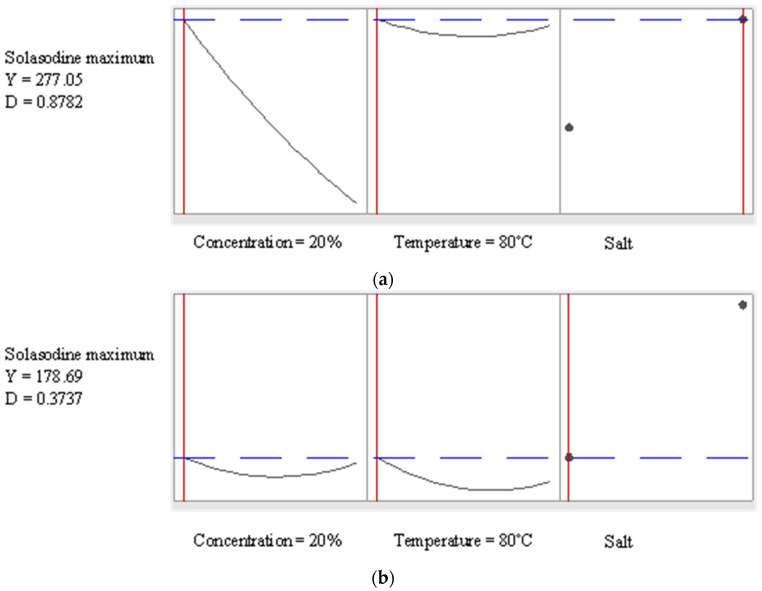
Optimal conditions for extraction of solasodine in the presence of (**a**) NaCl and (**b**) Na_2_CO_3_.

The response surface plots in Figure 7 demonstrated that as the concentration of salt was increased, the yield of solasodine decreased in the presence of Na_2_CO_3_ and this is consistent with the observations from the Box plots in Figure 4b. This also concurs with observations from the Pareto chart, Figure 2, which indicated the significant (*p* < 0.05) linear effect of the concentration of salt on the extraction of solasodine. In Figure 8a,b the predicted optimal extraction of solasodine in the presence of NaCl and Na_2_CO_3_ was 178.69 mg kg^−1^ and 277.05 mg kg^−1^, with a desirability score of 0.37 and 0.87, respectively. The desirability score of Na_2_CO_3_ was closer to one compared to NaCl. The closeness of Na_2_CO_3_ to the target requirement of one indicated the better reliability of the optimums obtained for maximal extraction of solasodine when Na_2_CO_3_ was used as an extraction agent. Additionally, comparisons of the maximal concentrations of solasodine obtained in Table 1, Figure 7 and Figure 8, indicated that as lower concentrations of Na_2_CO_3_ were used, the extraction of solasodine was enhanced compared to NaCl. Based on a previous study by Mokgehle et al. [20], the optimized method for extraction of solasodine was performed using a 30% salt solution, which resulted in the high enrichment of solasodine. However, the result herein (Figure 7) shows that above the 30% concentration, degradation of solasodine occurs. Therefore, higher salt concentrations contribute negatively to the extraction of solasodine, most probably due to salt-mediated hydrolysis [42].

## 4. Conclusions

The application of pressurized hot water-assisted aqueous two-phase extraction using a chaotrope and kosmotrope has proved to be a viable technique for extracting a pharmaceutically important metabolite, solasodine, from *S. mauritianum*. The optimized conditions for the extraction of solasodine in the presence of NaCl and Na_2_CO_3_ were a temperature of 80 °C at a salt concentration of 20%. The maximal experimental extraction of solasodine was 300.79 mg kg^−1^ and 162.34 mg kg^−1^ for Na_2_CO_3_ and NaCl, respectively. Based on statistical information obtained, the linear and quadratic effects of the concentration of salt (%), particularly for Na_2_CO_3_, had a significant effect (*p* < 0.05) on the extraction of solasodine during PHW-ATPE. The temperature, on the other hand, and other paired factors had an insignificant effect (*p* > 0.05) on the extraction of solasodine. The charge density on the CO_3_^2−^ ion was responsible for the greater salting-out ability of solasodine compared to Cl-, making Na_2_CO_3_ a better extractor. The extraction of solasodine from *S. mauritianum* could potentially be improved by applying miniaturized methods or even greener solvents such as deep eutectic solvents (DES).

## Figures and Tables

**Table 1:** Experiments designed by CCD for ATPE optimization with the respective responses and predicted values.

	Factor 1	Factor 2	Factor 3	Solasodine (mg kg^−1^)			
Run 1	% Salt	Temperature (°C)	Salt Type	Run 1	Run 2	Mean ± SD	Predicted
1	20	80	NaCl	149.421	175.273	162.34 ± 18	144.95
2	35	80	NaCl	164.782	152.101	158.44 ± 9	141.47
3	50	80	NaCl	167.337	173.475	170.40 ± 4	152.15
4	20	80	Na_2_CO_3_	276.235	325.356	300.79 ± 34	268.57
5	35	80	Na_2_CO_3_	137.631	242.474	190.05 ± 74	169.69
6	50	80	Na_2_CO_3_	90.256	121.386	105.82 ± 22	94.48
7	20	140	NaCl	145.755	147.766	146.76 ± 1	131.04
8	35	140	NaCl	133.142	145.833	139.48 ± 9	124.54
9	35	140	NaCl	141.55	185.129	163.33 ± 31	145.84
10	35	140	NaCl	143.837	193.206	168.52 ± 35	150.47
11	35	140	NaCl	153.087	193.257	173.17 ± 28	154.62
12	35	140	NaCl	160.403	181.400	170.90 ± 15	152.59
13	35	140	NaCl	147.844	189.184	168.51 ± 29	150.46
14	35	140	NaCl	133.210	189.690	161.45 ± 40	144.15
15	35	140	NaCl	146.783	199.122	172.95 ± 37	154.42
16	35	140	NaCl	144.264	145.378	144.82 ± 1	129.30
17	35	140	NaCl	135.107	156.155	145.63 ± 15	130.03
18	50	140	NaCl	144.261	150.600	147.43 ± 5	131.63
19	20	140	Na_2_CO_3_	231.064	288.117	259.59 ± 40	231.78
20	35	140	Na_2_CO_3_	132.953	148.042	140.49 ± 11	125.44
21	35	140	Na_2_CO_3_	143.273	174.470	158.87 ± 22	141.85
22	35	140	Na_2_CO_3_	140.993	178.487	159.74 ± 27	142.63
23	35	140	Na_2_CO_3_	144.551	183.655	164.10 ± 28	146.52
24	35	140	Na_2_CO_3_	160.268	135.875	148.07 ± 17	132.21
25	35	140	Na_2_CO_3_	175.495	187.357	181.42 ± 8	161.99
26	35	140	Na_2_CO_3_	182.617	177.587	180.10 ± 4	160.81
27	35	140	Na_2_CO_3_	138.686	209.858	174.27 ± 50	155.60
28	35	140	Na_2_CO_3_	157.500	173.412	165.45 ± 11	147.73
29	35	140	Na_2_CO_3_	161.165	191.737	176.45 ± 21	157.55
30	50	140	Na_2_CO_LL_	139.682	152.258	145.97 ± 9	130.33
31	20	200	NaCl	183.968	110.836	147.40 ± 52	131.61
32	35	200	NaCl	176.443	159.208	167.82 ± 12	149.84
33	50	200	NaCl	178.835	157.696	168.26 ± 15	150.24
34	20	200	N_a2_CO_3_	295.727	289.355	292.54 ± 5	261.20
35	35	200	Na_2_CO_3_	175.636	200.448	188.04 ± 18	167.89
36	50	200	Na_2_CO_3_	142.454	138.034	140.24 ± 3	125.22

## Data Availability

Not applicable.

## Sample Availability

Samples are available from the authors.
